# Anticipatory Modulation of Digit Placement for Grasp Control Is Affected by Parkinson's Disease

**DOI:** 10.1371/journal.pone.0009184

**Published:** 2010-02-12

**Authors:** Jamie R. Lukos, Dongpyo Lee, Howard Poizner, Marco Santello

**Affiliations:** 1 Department of Kinesiology, Arizona State University, Tempe, Arizona, United States of America; 2 School of Biological and Health Systems Engineering, Arizona State University, Tempe, Arizona, United States of America; 3 Institute for Neural Computation, University of California, San Diego, La Jolla, California, United States of America; 4 Department of Cognitive Science, University of California, San Diego, La Jolla, California, United States of America; Baylor College of Medicine, United States of America

## Abstract

**Background:**

Successful object manipulation relies on the ability to form and retrieve sensorimotor memories of digit forces and positions used in previous object lifts. Past studies of patients affected by Parkinson's disease (PD) have revealed that the basal ganglia play a crucial role in the acquisition and/or retrieval of sensorimotor memories for grasp control. Whereas it is known that PD impairs anticipatory control of digit forces during grasp, learning deficits associated with the planning of digit placement have yet to be explored. This question is motivated by recent work in healthy subjects revealing that anticipatory control of digit placement plays a crucial role for successful manipulation.

**Methodology/Principal Findings:**

We asked ten PD patients off medication and ten age-matched controls to reach, grasp and lift an object whose center of mass (CM) was on the left, right or center. The only task requirement was to minimize object roll during lift. The CM remained the same across consecutive trials (blocked condition) or was altered from trial to trial (random condition). We hypothesized that impairment of the basal ganglia-thalamo-cortical circuits in PD patients would reduce their ability to anticipate digit placement appropriate to the CM location. Consequently, we predicted that PD patients would exhibit similar digit placement in the blocked vs. random conditions and produce larger peak object rolls than that of control subjects. In the blocked condition, PD patients exhibited significantly weaker modulation of fingertip contact points to CM location and larger object roll than controls (*p*<0.05 and *p*<0.01, respectively). Nevertheless, both controls and PD patients minimized object roll more in the blocked than in the random condition (*p*<0.01).

**Conclusions/Significance:**

Our findings indicate that, even though PD patients may have a residual ability of anticipatory control of digit contact points and forces, they fail to implement a motor plan with the same degree of effectiveness as controls. We conclude that intact basal ganglia-thalamo-cortical circuits are necessary for successful sensorimotor learning of both grasp kinematics and kinetics required for dexterous hand-object interactions.

## Introduction

Skilled object manipulation is learned through practice, leading to the formation of sensorimotor memories for grasp control (e.g., [1]–[3]). Sensorimotor memories are used to associate previously experienced object properties (mass, texture, weight distribution, etc.) with the digit forces appropriate for manipulation [1], [3]–[9]. Importantly, they are involved in anticipatory grasp control, i.e., for planning grasp *before* the onset of the manipulation (e.g., [1]; for review see [10]). The advantage of anticipatory control is that it bypasses feedback delays associated with reflex-driven force adjustments triggered by object slip or tilt [2], [11]–[12], and therefore facilitates grasp stability.

Basal ganglia-thalamo-cortical circuits are known to be important for control of precision grip (for recent review, see [13]). Significant insight on the role of basal ganglia for grasp control has been gained through studies of patients affected by Parkinson's disease (PD) (e.g., [14]–[18]). Numerous studies have found that PD results in deficits in the coordination of digit forces during grasping (e.g., [19]–[23]) as well as impairments in the acquisition and/or retrieval of sensorimotor memories of manipulative forces [24].

A common feature of studies of grasping in PD is the use of grip devices that constrain digit placement on force sensors at fixed locations on the object, thus preventing subjects from choosing digit placement. However, the ability to change digit placement according to object properties is a fundamental component of skilled manipulation [25]–[26]. Digit placement on an object must be secured *before* forces can be generated to lift the object.

Anticipatory grasp control relies on the coordination of digit placement *and* forces [27]–[31]. It has recently been shown that subjects generate a given digit force distribution according to their choice of digit placement on a grasped object [30], [31]. As the first grasp event is contact, successful manipulation can only occur if subjects are able to plan digit placement on the object such that coordinated forces can be generated during grasp. Although this coordination has been documented in healthy individuals, the effect of PD on the ability to learn to anticipate appropriate digit position during grasp has yet to be examined. Unlike forces, subjects rarely change digit placement after contact [32]. Thus, appropriate planning prior to grasp is critical to ensure a successful manipulation. As the basal ganglia are important components of the neural networks involved in planning and anticipatory motor control [33], it is important to understand how these brain structures are involved in the planning of this crucial grasp component.

It should be emphasized that sensorimotor memories associated with the planning of fingertip placement and forces appear to involve different cortical regions [34] and be retrieved through independent processes [35]. Therefore, the known deficits exhibited by PD patients in using sensorimotor memories of digit forces might not generalize to anticipatory control of digit placement. Additionally, a task involving the choice of fingertip placement also has the potential to be more challenging than a task constraining digit placement. This is because the retrieval and use of sensorimotor memories associated with *both* grasp kinematics and kinetics may increase the computational load required for anticipatory grasp coordination.

When healthy subjects can predict an object's center of mass (CM) through previous object lifts, they learn to modulate digit positions on the object in an anticipatory fashion [29]. The present study was designed to determine whether PD affects this anticipatory control of digit placement. Furthermore, we quantified the magnitude of object roll during lift to infer the extent to which digit forces were appropriately modulated to object CM. Similar to the task used by Lukos et al. [29], [35], we asked subjects to grasp, lift, hold and replace an object whose CM either remained the same across consecutive trials (blocked condition), or was altered on a trial-to-trial basis (random condition). The only task requirement was to minimize object roll during lift.

We hypothesized that impairment of the basal ganglia-thalamo-cortical circuits in PD patients in a dopamine-depleted state would reduce their ability to use sensorimotor memories to anticipate appropriate digit placement as a function of object CM. This deficit would be revealed by PD patients exhibiting similar digit placement in the blocked vs. random conditions and produce larger peak object rolls than that of control subjects.

## Methods

### Subjects

Ten subjects with idiopathic PD (7 males, 3 female; mean age 68.6 years, range 50–82 years) and ten healthy age-matched controls (6 males, 4 females; mean age 68.2 years, range 50–77 years) participated in this experiment. A post hoc power analysis (G*Power3 software; [36]) on our behavioral measure, peak object roll (see below), yielded an adequate effect size (*d* = 0.667) and sample size [power (1-β) >0.80; α = 0.5] for each group. The protocols were approved by the University of California, San Diego Institutional Review Board and the experiment was conducted according to the Declaration of Helsinki. Written informed consent was obtained for all subjects. Nineteen of the 20 subjects were right handed as measured by the Oldfield Handedness Questionnaire [37]. The remaining subject, a control subject, was ambidextrous. Subjects with PD were all mild to moderate in degree of symptoms ([38], stage 2 and 3). They had experienced symptoms of PD from 6–25 years, having a mean disease duration of 11.9 years. Early stage PD patients were tested to assure that they could perform the task as required (see *Experimental task* below), and that any between-group differences resulted from impairments of the basal ganglia circuitry rather than from advanced sensorimotor and/or cognitive deficits associated with later stages of the disease [33].

Table 1 provides a description of the clinical characteristics of each subject, all of whom were referred and screened by a neurologist to exclude those with marked dyskinesia, tremor, or ON/OFF medication fluctuations that would prevent performance of the experimental grasping task or confound interpretation of the acquired kinematic data. PD subjects were also screened to exclude patients with depression or dementia using the Beck Depression Inventory and the Mini-Mental State Examination. Any patients having additional deficits in other neural systems (“Parkinson plus” patients) were excluded. The exclusion criteria for all subjects (PD and age-matched controls) were: (1) arthritis in the dominant upper extremity, (2) orthopedic or visual problems that would interfere with the task, (3) upper extremity weakness that would prevent task performance, and (4) other co-existing neurological or psychiatric disease. Patients were studied in the ‘OFF’ state, in the morning before receiving medication that day, being at least 12 hours off medication [39]. The rationale for testing patients in a dopamine depleted state (off medication) was to quantify the effects of more pure basal ganglia impairment on anticipatory grasp control than would be the case with medicated patients.

**Table 1:** Clinical characteristics of Parkinson's disease patients and age-matched of controls.

Patient ID	Age (years)	Sex	Hand-edness	Disease duration (years)	UPDRS	Action Tremor Score	H & Y Stage	Medications	Control ID	Age (years)
1PD	50	M	R	8	59.5	0	3	Lev; LevR; Sel; Ent	1C	50
2PD	63	F	R	10	22.0	0	2	Lev; Pr; Pr	2C	63
3PD	66	F	R	10	33.5	1	2	Pr; Sel; Am	3C	66
4PD	67	M	R	9	41.5	1	2	Lev; Ent; Art	4C	69
5PD	68	F	R	11	46.5	0	3	LevR; Pr; Ent; Ras	5C	69
6PD	71	M	R	25	55.5*	1	3	LevR; Ent; Sel; Pr; Am	6C	71
7PD	71	M	R	15	34.0	1	3	LevR; Pr; Am	7C	71
8PD	72	M	R	15	38.5	1	2	St; Sel; Rop	8C	73
9PD	76	M	R	10	34.5	1	2	Lev; Pr	9C	73
10PD	82	M	R	6	42.0	1	3	Lev; LevR; Sel; Am	10C	77

The table shows the clinical characteristics of the Parkinson's disease patients. Duration is years since first remembered parkinsonian symptom. UPDRS: United Parkinson's Disease Rating Scale, Motor section (range from 0–108). Higher scores indicate greater impairments. UPDRS score prorated to scale ending at 108. Medication codes are as follows: LevR (Carbidopa/levodopa sustained release); Lev (Carbidopa/levodopa, regular formulation); Pr (Pramipexole); Sel (Selegiline); Ent (Entacapone); Br (Bromocriptine); Rop (Ropinirole); St (Stalevo (Carbidopa/levodopa/entacapone)); Ras (Rasagiline); Am (Amantadine); Rot (Rotigotine); Art (Artane (trihexyphenidyl)). *: left arm could not be tested due to injury.

### Experimental Task

**Figure 1:**
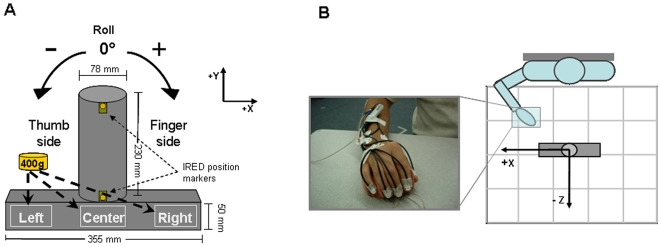
Experimental setup. Panel A shows the object (frontal plane) used for our task and the slots where a mass was added to change object CM location. Object rolls towards the thumb and finger sides were defined as negative and positive angles, respectively, relative to the vertical (*y*) in the gravitational frame of reference. Note that the view of the object is shown from the subject's perspective. IRED markers were placed along the midline of the cylinder on the back of the object and therefore did not come into contact with the digits during the grasp. Panel B shows a photo of the hand at the starting position outfitted with IRED markers on the fingertips, hand, radius and ulna as well as a diagram of the workspace of the task (top view; figure is not to scale).

We asked subjects to reach, grasp, lift and replace a cylindrical object with their right hand. The task and the object used for the present study are the same as used in two previous reports with healthy, young subjects [29], [35]. The object consisted of a cylinder made of sturdy cardboard covered with black matte tape for uniform texture mounted on a wooden rectangular base (Fig. 1A). The cylinder was aligned with the subject's midline (Fig. 1B). The distance between the start position of the hand and the center of the cylinder on the *xz* plane was at a comfortable reaching position ∼30 cm away from the subject. The object's center of mass (CM) was changed by adding a mass (0.4 Kg) in the base of the object in one of three slots: left, center, or right, thus creating an external torque of −0.55 N⋅m, 0 Nm, and +0.55 N⋅m, respectively (Fig. 1A). The total mass of the object with the added mass was 0.81 Kg.

We asked subjects to perform this task by using the fingertips of all digits, but no instructions were given on where to grasp the object along the cylinder. The only task requirement was to keep the object as vertical as possible (i.e., minimize object roll in the *x-y* plane) while lifting the object ∼15–20 cm above the table. Subjects were informed that object CM was going to remain the same across blocks of trials (blocked condition) or be changed in a pseudo-randomized order from trial to trial (random condition). The blocked condition was used to quantify the time course of the adaptation of digit placement and forces (see below) as a result of repeated manipulation. The random condition was used to prevent planning of digit contact points and forces as a function of object CM location, since implicit knowledge of CM gained from trial *n* could not be used on trial *n+1*. Subjects' ability to plan digit forces was quantified indirectly by measuring peak object roll (see [29] for more details).

One of the experimenters demonstrated the grasp, lift and replace task before asking the subject to perform one practice trial (center CM). Subjects were instructed to perform the task at a self-selected speed but as a single, smooth movement to prevent the gaining of information about CM location by stopping the movement shortly after object lift onset. Offline analyses revealed that all but one subject were able to follow our task instructions. PD patient #10 (see Table 1) frequently performed several sub-movements during the object lift. Therefore this patient and his age-matched control were eliminated from statistical analyses.

Subjects performed a total of 32 trials for both the random and blocked conditions, i.e., 12 trials for left CM, 12 trials for right CM, and 8 trials for center CM, for a total of 64 trials per experiment. In the blocked condition, the presentation of each CM block of trials was counterbalanced across subjects. The randomization scheme was designed to present the same number of right and left CM trials (*n* = 12) but a smaller number of center CM trials (*n* = 8). The rationale for using center CM trials was to increase the number of CM combinations throughout the random trial series, hence weakening subjects' ability to predict the CM trial sequence. However, unlike the left or right CM, the center CM does not generate a torque on the object. As the center CM does not challenge subjects' ability to minimize object roll during the lift, only the data associated with left and right CM were analyzed.

Within each experiment, half of the subjects started with the blocked condition followed by the random condition and vice versa for the remaining subjects. The presentation of object CM was designed such that subjects never experienced the same CM location when transitioning from one experimental condition to another. The weight exchanges were performed out of subjects' view. The location of the added mass was blocked from view by a wooden board placed on the front side of the object facing the subject during the entirety of the experiment (Fig. 1A).

### Data Recording

Movement of the hand and object was recorded using an active marker 3D motion capture system (PhaseSpace, Inc., San Leandro, CA; frame rate: 240 Hz). Twelve cameras were placed in a semi-circle 1–2.5 m from the subject, who was seated at a table where the object was placed (Fig. 1B). We calibrated the system prior to each data collection. Subjects were outfitted with light-weight infrared emitting diode (IRED) active markers (5 mm in diameter) on the center of the fingernail of each digit, on the back of the hand (approximately on the center of the second metacarpal bone), on the styloid processes of radius and ulna, on the lateral epycondile of the humerus and on the lateral aspect of the shoulder joint. We verified that placement of the IRED markers did not prevent motion of the digits and/or the wrist by asking subjects to fully flex and extend all digits as well as to grasp the object prior to the start of the experiment. The spatial accuracy of the recording system was ∼1 mm in the *x*, *y* and *z* planes, and its resolution was 0.1 mm.

As described in our previous work [29], [35], analysis of hand kinematics focuses on the spatial distribution of digit contact points as defined by the marker placed on the center of each fingernail. We also placed IRED markers on the top and bottom of the cylinder to measure object kinematics and the spatial distribution of the fingertips relative to the object (see below). The behavioral performance of the task – peak object roll – was used as an indirect measure of anticipatory force control, with smaller rolls being evidence of more accurate digit force scaling to the expected external torque following onset of object lift (e.g., [5], [6]; see [29] for more details). Note that using force sensors at fixed locations on the object to directly measure individual digit forces was not feasible since our protocol was designed to let subjects choose placement of all digits.

### Data Processing

Each trial was manually inspected to verify proper marker identification and the absence of movement artifacts. These data were then run through a 4^th^ order low-pass Butterworth filter with a 10 Hz cutoff.

#### Digit kinematics

For the analysis of fingertip location, we measured the anteroposterior and vertical coordinates (*z*-axis and *y*-axis, respectively; see spatial frames of reference in Fig. 1) of fingertip position at the time of contact with the object. We defined *contact time* as the time at which the tangential velocity computed in three dimensions of the marker on the tip of each digit reached its minimum value between the time of peak wrist velocity (defined by a marker on the radius) and object lift onset (defined below). Accuracy of this algorithm was verified offline by using measures computed from the object model (cylinder diameter) and the hand model (distance between the thumb and the digits). We found that fingertip tangential velocity accurately defined the time of contact between the fingertip and the object (see [29] for details). We defined *contact points* as the anteroposterior and vertical coordinates of fingertip location at contact time. We then transformed the *y*- and *z*-coordinates of each contact point to an object-centered frame of reference by expressing them relative to the *y*- and *z*-coordinates of the center of the base of the cylinder (0,0).

#### Object kinematics

We measured five variables: (1) *object roll*, (2) *object lift onset*, (3) *time to peak object roll*, (4) *object vertical velocity*, and (5) *object lift duration*.

1. *object roll* in the *xy* plane (Fig. 1A) was defined as the angle between the gravitational vertical and the line connecting top and bottom markers on the cylinder. We measured peak object roll occurring within the lift duration (see below). In most trials, peak object rolls (ranging from ∼ −22.3° to +27.2° for left and right CM, respectively) occurred at reaction time latencies, i.e., ∼120–170 ms after object lift onset. Our behavioral analysis focused on the initial peak roll to quantitatively assess *anticipatory* control mechanisms. Correct identification of peak object roll was verified offline for each trial (see [29] for more details).
2. *object lift onset* was defined at the time at which the tangential velocity of the radius marker crossed a threshold (1 mm/s) and remained above it for longer than 500 ms.
3. *time to peak roll* was defined as the latency between object lift onset and the time at which peak roll occurred. This variable was analyzed to quantify how long subjects took to generate adequate forces to counteract object roll, hence a measure of reaction time.
4. *object vertical velocity* was computed as the derivative of the position of the *y*-coordinate of the marker on the top of the object (Fig. 1A). Peak object vertical velocity was computed (a) between onset and end of lift and (b) between onset of lift and peak object roll. Peak object vertical velocity from object lift onset to end was computed to detect overall differences between controls and PD in the speed at which the object was lifted, the expectation being that PD patients would lift the object slower than controls. Peak object vertical velocity between lift onset and peak object roll was computed to determine whether the initial speed of object lift, which could have affected peak object roll, differed among groups and experimental conditions.
5. *object lift duration* was defined as the time between object lift onset (when the tangential velocity of the top center marker of the object crossed a velocity threshold of 5 mm/s and remained above it for longer than 200 ms) and object lift end (when the top center marker of the object reached its peak height).

#### Reach and grasp temporal variables

Planning the positioning of the fingertips on desired locations on the object requires accurate transport of the hand during the reach. Therefore, we computed the following variables associated with arm kinematics:

1. *reach onset* was defined as the time at which the tangential velocity of the radius marker crossed a threshold (2 mm/s) and remained above it for longer than 200 ms;
2. *reach duration* was defined as the time interval between reach onset and object lift onset (see below);
3. *contact duration* was defined as the time interval between the first and last digit contacting the object (as measured by the contact time algorithm stated above);
4. *pre-lift duration* was defined as the time interval between the last digit contacting the object and object lift onset.

We wrote custom software (Matlab, The MathWorks, Natick, MA) to compute all of the above variables.

### Statistical Analyses

We performed a between-group (control vs. PD) multivariate repeated measures ANOVA with “Predictability” (blocked vs. random) and “CM Location” (left vs. right) as within-subject factors on the vertical and anterior-posterior contact points of all five digits. We also performed a between-group (control vs. PD) repeated measures ANOVA with “Predictability” (blocked vs. random) as a within-subject factor, with data from left and right CM trials averaged for each condition on the absolute value of peak object roll (the rationale being explained in [29]). Furthermore, an analysis of individual trial performance was performed to describe the time course of the learning associated with blocked presentation of object CM as well as possible differences in learning rates between groups. For these analyses we used a between-group (controls vs. PD) repeated measures ANOVA with “Trial” (12 levels) as the within-subject factor.

The temporal variables of *(a)* reach duration, *(b)* contact duration, *(c)* pre-lift duration, *(d)* time to peak object roll, *(e)* peak object vertical velocity during lift, *(f)* peak object vertical velocity prior to peak object roll, and *(g)* lift duration were also explored with between-group repeated measures ANOVAs to assess any grasp timing differences between groups.

Post hoc *t*-tests were performed for significant main effects that warranted further exploration of comparisons of interest with Bonferroni corrections (α-level of *p*≤0.05) applied. All data are reported as mean ± standard error (S.E.).

## Results

### Spatial Distribution of Digit Contact Points

We found that the maximum modulation of contact points was ∼2.5 times greater on the vertical (*y*-axis) than on the anterior-posterior (*z*-axis) dimension (Fig. 1), i.e., 13.8 mm and 5.5 mm, respectively. We therefore present all analyses of digit contact points in the vertical dimension only.

**Figure 2:**
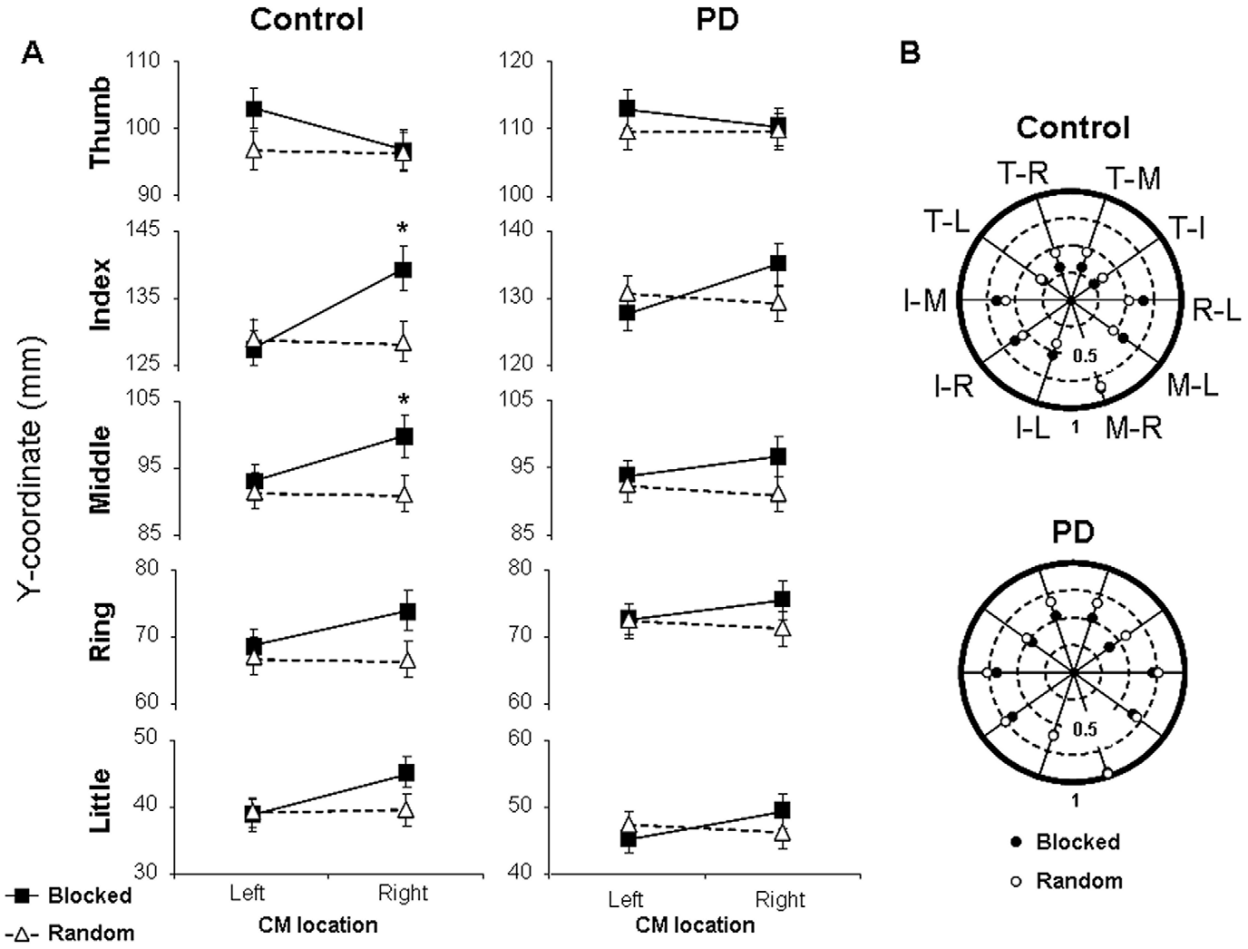
Individual fingertip contact points and covariation of digit pair contact points. Panel A depicts fingertip vertical location relative to the base of the cylinder for each digit as a function of CM location and predictability condition for controls and PD patients (left and right columns, respectively). The scale of the vertical axes is the same for all plots to allow comparison across digits. Data are means ± SE of all subjects. Asterisks denote a significant difference at *p*<0.05. Panel B represents the magnitude of the correlation coefficient (Pearson's *r*) of digit pair contact points for the controls and PD patients (top and bottom polar plots, respectively). The *r*-values shown in each polar plot were averaged across all subjects (after z-normalization). Values nearing zero, i.e., closer to the center of the polar plot, represent greater independence of digit pair contact points than values nearing one. White and black circles denote random and blocked conditions, respectively. T, I, M, R and L denote thumb, index, middle, ring and little fingers, respectively. Note that for digit pair correlations with similar values for blocked and random, it may appear as a single white circle.

As expected, controls exhibited clear differences in the modulation of fingertip contact points to object CM in the blocked vs. random condition. Specifically, in the blocked condition controls placed the thumb higher for left than right CM locations, whereas an opposite pattern was found for the fingers. This is consistent with our previous work on healthy young subjects [29]. PD patients also modulated fingertip contact points to object CM location in the blocked condition, but such modulation was not as clear as controls (Fig. 2A). In the random condition, both subject groups used a similar distribution of contact points across trials regardless of object CM location.

The above observations were confirmed by multivariate analysis revealing significant interactions CM Location × Group and CM Location × Predictability, as well as a main effect of Group, CM Location, and Predictability ([*F*_(5, 180)_  = 2.52, 21.69, 36.58, 16.61, 6.96, respectively]; all *p*<0.05). The between-subject effect of Group was found for the thumb and little finger contact points ([*F*_(1,184)_  = 11.57 and 5.24, respectively]; both *p*<0.01). The main effect of CM Location was found for the thumb, index, and little finger contact points ([*F*_(1,184)_  = 5.88, 17.12, and 6.88, respectively]; all *p*<0.05) and a main effect of Predictability was present for all digits but little ([*F*_(1,184)_  = 4.09, 6.90, 12.12 and 7.91 for thumb, index, middle, and ring, respectively]; all *p*<0.05). Lastly, the CM Location × Predictability interaction was present for all digits ([*F*_(1,184)_  = 4.35, 26.71, 7.74, 6.21, and 8.51 for thumb, index, middle, ring, and little, respectively]; all *p*<0.05).

The post hoc analysis revealed a significant difference between blocked and random conditions in the control group for the index and middle fingers when the CM was on the right (Fig. 2A, 1^st^ column, *p*<0.01). There was also a significant difference between left and right CM locations in the blocked condition for the index finger (*p*<0.01). No significant modulation of contact points to object CM location was found within the PD group in the blocked vs. random conditions (Fig. 2A, 2^nd^ column).

The weaker modulation of contact points exhibited by PD patients in the blocked condition could have been due to action tremor. However, the PD patients had little to no action tremor: the scores from UPDRS tested off medication were 0 or 1, reflecting either no or mild action tremor (see Table 1). To further rule out action tremor as a potential contributor to the above group differences in digit placement, we tested the difference in each digit contact point (a) variability and (b) modulation to object CM between patients with no vs. mild action tremor (3 vs. 6 patients, respectively). No significant difference was found between these two PD sub-groups (*p*>0.05).

### Linear Correlation between Contact Points

In healthy young controls, we previously found greater independence between digit placement was present in the blocked compared to the random condition, especially for thumb-finger pairs [29], [35]. Here we performed the same analysis (linear regression) to assess whether PD and controls differed in the coordination of contact points as a function of experimental condition (Fig. 2B). We found that the controls tended to exhibit greater independence of contact points than PD patients, i.e., *r*-values between digit pairs were smaller for controls than PD patients (average r-values of 0.51±0.03 vs. 0.64±0.03, respectively). However, differences between predictability conditions for both groups were not as clear as that previously reported on healthy young subjects, indicating that the older controls as well as the PD patients show less independence of contact points than young adults.

### Peak Object Roll

**Figure 3:**
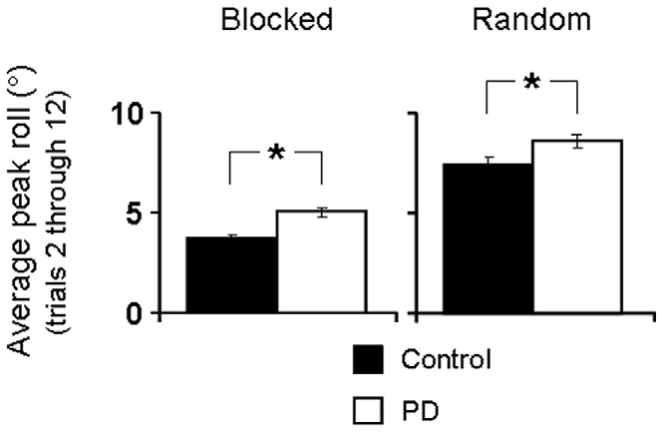
Peak object roll. Peak object roll averaged throughout the blocked (right plot) and random (left plot) trial sequence for controls and PD patients. Asterisks denote a significant difference at *p*<0.05.

Peak object roll is an indirect measure of anticipatory control of digit forces and task performance (see Methods). Figure 3 shows peak object roll averaged from trial 2 through 12 (note that the first trial is omitted from analyses since subjects cannot yet anticipate CM location).

We found evidence of anticipatory force control planning in both groups, as control and PD subjects showed smaller object rolls in the blocked compared to random conditions (main effect of Predictability: F_(1,195)_  = 243.20, *p*<0.01; no significant interaction Group × Predictability, *p*>0.05). This was confirmed by post hoc analyses (*p*<0.01; Fig. 3). This finding indicates that PD patients benefited from the blocked trial presentation of CM location as their anticipatory digit force control improved with repeated lifts (see also ‘trial-to-trial learning’ below). However, control subjects were able to minimize object roll to a greater extent than PD patients in both the blocked and random conditions (main effect of Group: F_(1,195)_  = 16.18, *p*<0.01; Fig. 3), indicating that anticipatory control in PD subjects was inferior to that exhibited by control subjects.

### Time to Peak Object Roll

Time to peak object roll is a measure of how quickly a corrective response to the external torque can be initiated after object lift onset. Subjects in both groups were able to initiate a corrective response faster in the blocked than in the random condition (122±3 and 152±3 ms, respectively; main effect of Predictability: [*F*_(1,195)_  = 60.38], *p*<0.01; no main effect of Group, *p*>0.05).

**Figure 4:**
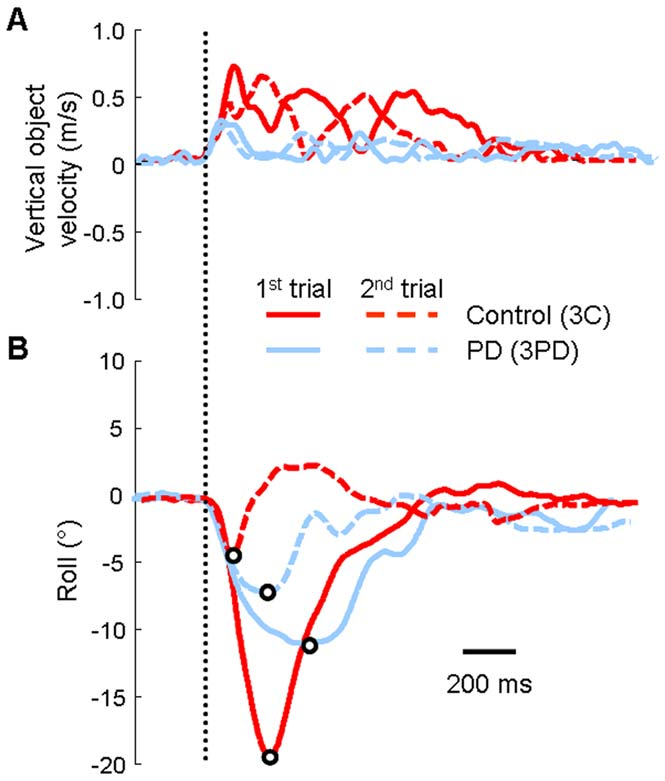
Object kinematics. Panel A presents vertical object velocity vs. time during object lift. Panel B shows object roll vs. time during object lift. The open circles denote peak object roll. Both panels are data of one subject with PD and a control subject (subjects 3PD and 3C, respectively, see Table 1) for the first two trials of the blocked condition. The solid traces represent the first trial for each subject and the dashed traces represent the second trial. The vertical dotted line represents object lift onset.

Figures 4A and B show the time course of object vertical velocity during lift and object roll during the first two trials of the blocked condition for one PD patient and an age-matched control. The peak object vertical velocity during lift was lower for the PD patient than the control on the first and second trial. However, peak vertical velocity prior to peak object roll was comparable between the two representative subjects in the 2^nd^ trial (see first peak of dashed lines, Fig. 4A). Note that peak object roll associated with the first trial was larger for this control than for the PD patient. However, the control was able to reduce peak object roll on the second trial to a greater extent than the PD patient (Fig. 4B).

We found a significant between-group difference in peak vertical object velocity during lift ([*F*_(1,195)_  = 9.40], *p*<0.01). Controls lifted the object at a higher velocity than PD patients in the blocked condition (0.61±0.03 m/s vs. 0.54±0.02 m/s, respectively) and in the random condition (0.62±0.02 m/s vs. 0.50±0.02 m/s, respectively; *p*<0.05 for all post hoc comparisons). However, assessment of peak vertical object velocity prior to peak object roll showed no between-group difference (controls: 0.34±0.02 m/s and 0.31±0.02 m/s and PD: 0.34±0.02 m/s and 0.35±0.02 m/s, *p*>0.05). Therefore, lack of between-group differences in vertical object peak velocity prior to peak roll fails to explain the above significant group differences in the magnitude of peak roll (Fig. 3).

Lastly, no group differences were found in lift duration, with both groups exhibiting longer lift durations during the random compared to blocked condition (main effect of Predictability: [*F*_(1,195)_  = 50.09], *p*<0.01). Post hoc analyses revealed that this difference was significant for control subjects (502±21 and 420±15 ms, respectively) and PD patients (524±20 and 445±15 ms, respectively).

### Trial-to-Trial Learning of Object Roll Minimization

**Figure 5:**
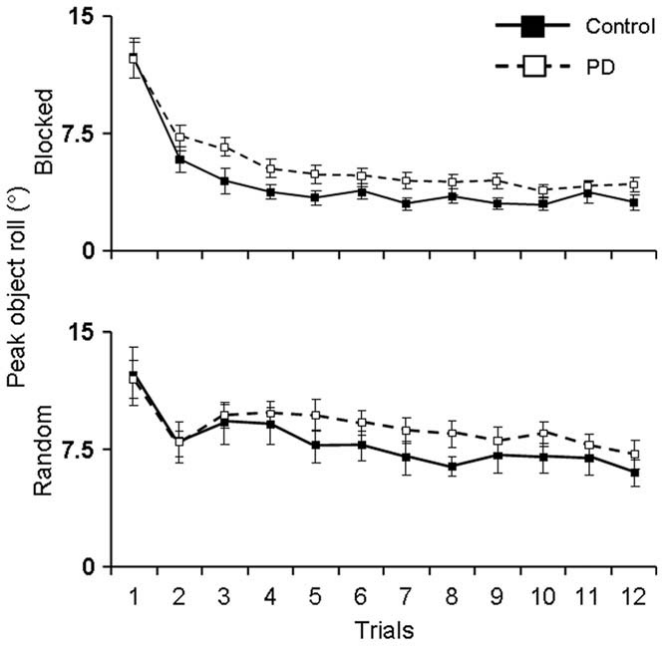
Performance curves of object roll minimization. Peak object roll averaged across trials 2 through 12 for controls and PD patients for the blocked (top plot) and random (bottom plot) conditions. Asterisks denote a significant difference at *p*<0.05.

It is clear from the analysis of average object roll that in the blocked condition both groups were able to learn to minimize object roll. However, the above analysis does not assess the trial-to-trial adaptation of object roll minimization. The performance curves for the control and PD groups presented in Figure 5 show that peak object roll is consistently greater for the PD subjects on every trial, indicating that PD patients did not learn to minimize object roll to the same extent as controls throughout the experiment. However, the performance curves run nearly parallel as a function of trial, thus suggesting that the rate of learning object roll minimization may be similar in both subject groups (but see below).

As expected, peak object roll changed significantly as a function of trial (main effect of Trial: F_(11,18)_  = 9.497, *p*<0.01). The overall rate at which PD patients and controls learned how to minimize peak object roll was similar (no significant interaction Trial × Group, *p*>0.05). Post hoc analyses of blocked condition trials revealed significantly greater peak object rolls in the 1^st^ and 2^nd^ trials compared to the remainder of the trials in both groups (*p*<0.05). By the 3^rd^ trial, control subjects had learned to successfully minimize object roll for the remainder of the trial sequence, i.e., no significant difference in peak object roll from 3^rd^ trial onward (*p*>0.05). However, PD patients did not successfully learn to minimize object roll until the 4^th^ trial as significant differences between the 3^rd^ trial and remaining trials still existed (*p*<0.05). Therefore, although the difference in learning rates may be small (a single trial), this shows that PD patients need more experience in order to learn to minimize object roll compared to controls.

### Temporal Variables: Reach and Grasp

#### Reach duration

PD patients took longer during the blocked compared to the random condition (633±19 and 611±20 ms, respectively) whereas control subjects took less time to reach during the blocked than random condition (657±20 and 674±20 ms respectively; significant interaction Predictability × Group: [*F*_(1,195)_  = 4.24], *p*<0.05). However, post hoc test revealed no significant differences between groups or predictability conditions.

#### Contact duration

This variable was longer for both subject groups in the blocked vs. random condition, with controls exhibiting longer contact duration than PD patients (controls: 87±5 and 80±5 ms, respectively; PD: 68±5 and 54±5 ms, respectively). Both differences were statistically significant (main effect of Predictability: [*F*_(1,195)_  = 8.51], *p*<0.01; main effect of Group: [*F*_(1,195)_  = 14.69], *p*<0.01) and confirmed by post hoc tests (*p*<0.05 between groups and for both the blocked and random conditions).

#### Pre-lift duration

We found significant group differences in pre-lift duration ([*F*_(1,195)_  = 13.44], *p*<0.01) and between predictability conditions ([*F*_(1,195)_  = 8.26], *p*<0.01; controls: 38±3 and 31±3 ms and PD: 31±3 and 19±3 ms for blocked and random condition, respectively). Post hoc comparisons showed that a significant group difference existed, but was small (∼9.5 ms).

In summary, PD patients were not slower than controls during hand transport or in the grasp-to-lift coordination. This is consistent with previous observations of clinically bradykinetic PD patients who may show normal movement durations when reaching for 3D targets, particularly when full vision is available [40], [41].

## Discussion

### Sub-Optimal Use of Sensorimotor Memories for Anticipatory Modulation of Fingertip Contact Points and Forces in PD

As expected, when object CM location could be predicted through previous lifts, control subjects were able to exhibit anticipatory control of fingertip contact points during object grasp by modulating digit placement as a function of CM location. This behavior was also associated with a greater ability to minimize object roll, implying that control subjects distributed fingertip forces on the object according to the direction and magnitude of the external torque. In contrast, PD patients in a dopamine-depleted state failed to use sensorimotor memories to significantly modulate contact point when object CM location was predictable (Fig. 2). These results suggest that the basal ganglia circuitry play a significant role in the planning of digit placement for object manipulation.

Surprisingly, despite an impairment in the ability to modulate contact points to object CM location, PD patients were nevertheless able to minimize object roll to a greater extent for consecutive lifts of the same object CM than when it changed randomly across trials (Fig. 3). This finding points to a residual ability to generate, store and retrieve sensorimotor memories associated with previous object manipulations. This interpretation is consistent with the finding that the performance curves of control and PD subjects ran nearly parallel for both the blocked and random conditions (Fig. 5).

It should be noted, however, that control subjects were better than PD patients at minimizing object roll as shown by the production of significantly smaller object rolls on every trial in both the blocked and random conditions. The difference in performance between groups was small (on average 1.3°) which may have been a consequence of testing relatively mild PD patients. Nevertheless, the consistency with which controls outperformed PD patients and difference in rates at which peak object roll minimization was learned in the blocked condition (3^rd^ vs. 4^th^ trial for controls vs. PD patients, respectively) point to a deficit in the planning and/or execution of digit force sharing patterns. This deficit, coupled with weak anticipatory modulation of fingertip contact points to object CM, suggest that PD patients were unable to take advantage of sensorimotor memories gained from previous manipulations to the same extent as their age-matched controls. As action tremor and slowness of movement were ruled out as potential contributors to these group differences, our results are consistent with other studies describing the effect of dopamine modulation on the ability to accurately store spatial memories [42]. Our data provide insight about the sensorimotor memory deficits associated with dopamine depletion indicating that implicit learning of digit placement and forces during grasp are both affected when the basal ganglia-thalamo-cortical circuits are impaired.

### PD-Induced Deficits in the Coordination of Digit Placement and Forces

Although past studies have revealed deficits in the coordination of digit forces during grasping in PD (e.g., [19]–[23]), these studies have been performed with grip devices that prevented subjects from choosing digit position on the object. Our task is the first to examine the effects of PD on anticipatory grasp control during a natural task devoid of digit placement constraints. This is a crucial difference in experimental design, since the ability to change digit placement according to object properties is a fundamental component of skilled manipulation. Furthermore, we have previously reported that sensorimotor memories for digit position and forces appear to be processed independently in healthy young adults [35]. Repetitive transcranial magnetic stimulation (rTMS) studies have also shown that different cortical structures are responsible for digit position and forces [34] as well as use of explicit vs. implicit knowledge about object properties [43] for anticipatory grasp control. Therefore, previously reported deficits in anticipatory force control in PD might not have generalized to anticipatory control of digit placement on an object.

As our task requires careful planning of both digit forces and positions prior to object manipulation, one can argue that it allows subjects to use many, equally valid strategies to counteract the external moment generated by the added mass. Therefore, these task conditions could be easier than a task where the position of the digits is always the same as performed in previous studies of grasp control in PD (e.g., [22]–[24]) which limits the number of successful strategies. While this might be true for healthy individuals, anticipatory control of an additional variable (digit position) potentially increases the computational load associated with anticipatory grasp control in PD patients. For instance, trial-to-trial variability in digit position also requires a concurrent covariation of digit forces such that the same net digit moment is attained on each trial [30], [31].

We found that PD patients did not significantly modulate contact points whereas control subjects exhibited anticipatory control of digit placement. This suggests that the choice of contact points was not incorporated into the anticipatory grasp control strategy of PD patients. However, PD patients were able to minimize roll to a greater extent in the blocked compared to random conditions. Therefore, anticipatory control mechanisms were primarily used by PD patients in the force domain when object CM was predictable. In contrast to the present results, Muratori et al. [24] found no differences in the ability to minimize object roll between PD patients and controls in response to predictable changes in object CM using a grip device that constrained digit placement. However, our findings cannot be directly compared to those of Muratori et al. [24] due to differences in magnitude of object torque generated by the added mass. Nevertheless, these performance differences suggest that the additional choice of contact points in a grasping task may increase the computational load of the system for the generation and/or retrieval of sensorimotor memories. This may further challenge the ability of PD patients in a dopamine depleted state to effectively implement anticipatory grasp control.

### Anticipatory Control Deficits in PD

One strategy that subjects could have used to limit object roll might have been grasping the object with larger than necessary grip forces and/or stiffening the wrist. Since we could not measure digit forces (see *Methods* for rationale), we were unable to determine whether or to what extent such strategy was used. However, simply grasping the object harder might not result in smaller object rolls since object roll minimization requires the net digit torque to match the external torque (also note that the PD performance curves are not consistent with such strategy).

The above findings indicate that PD patients may have an implicit understanding of the anticipatory control of digit contact points and forces necessary to produce a compensatory torque at object lift onset. Nevertheless, consistent with previous observations of deficits in anticipatory multi-digit grasp force planning [23], PD subjects fail to implement such a plan with the same degree of effectiveness as controls. Performance differences between controls and PD patients do not appear to arise from cognitive impairments in understanding the task requirements, as indicated by the fact that PD patients learned to minimize object roll after three trials (top panel, Fig. 5). Therefore, behavioral deficits must be a result of inaccurate anticipatory control strategies generated from sensorimotor memories. Although it is unclear whether this impaired behavioral response is the result of deficits in motor planning, execution, or both, it is known that PD patients have deficits in sensorimotor integration [44]. PD patients are excessively dependent on visual information to effectively execute hand kinematics for reaching to 2D or 3D targets [45]–[47], preshaping their hands when reaching for and grasping objects of different shapes [17], and for coordination of arm and trunk movements [41]. Since PD patients show normal early proprioception-related EEG-potentials, but altered cortical processing of kinesthetic signals at longer latencies, their deficits appear to be alterations in central integrative processes [48], thus possibly affecting the storage and/or retrieval of sensorimotor memories.

### Conclusions

We have shown that PD patients are less able than age-matched controls to generate and/or use sensorimotor memories of digit placement and forces derived from repetitive object grasp and manipulation. Nevertheless, some degree of implicit learning was found as evidenced by PD patients' incremental ability to minimize peak object roll during consecutive object lifts. This suggests that, even though PD patients may have a residual ability of anticipatory control of digit contact points and forces, they fail to implement a motor plan with the same degree of effectiveness as controls. These results point to an involvement of basal ganglia circuitry in planning of accurate digit positioning prior to manipulation.
